# Population Genetics of *Bactrocera minax* (Diptera: Tephritidae) in China Based on *nad4* Gene Sequence

**DOI:** 10.3390/insects10080236

**Published:** 2019-08-02

**Authors:** Feng Hong, Lizhi Gao, Hong-Liang Han, Pan Wang, Jia Wang, Dong Wei, Yinghong Liu

**Affiliations:** 1 College of Agriculture, Xinyang Agriculture and Forestry University, Xinyang 464000, China; 2 Chongqing Key Laboratory of Entomology and Pest Control Engineering, College of Plant Protection, Southwest University, Chongqing 400715, China; 3 State Cultivation Base of Crop Stress Biology for Southern Mountainous Land, Academy of Agricultural Sciences, Southwest University, Chongqing 400715, China

**Keywords:** *Bactrocera minax*, mitochondrial DNA, genetic differentiation, phylogenetics

## Abstract

*Bactrocera minax* (Enderlein) (Diptera: Tephritidae) is an important citrus pest in Asia with a non-uniform distribution. In some locations, it had been reported to occur but was either eradicated or disappeared itself. To understand species dispersal of *B. minax*, we collected and analyzed 359 individuals from 18 localities in China. One mitochondrial DNA gene fragment (*nad4*) was used to investigate the genetic diversity and population genetic structure of *B. minax*. The populations were divided by phylogenetic analyses and statistical parsimony haplotype networks into three branches: a Central China (CC) branch, a Western China (WC) branch, and a Southern China (SC) branch. A total of 93 variable sites (15.6% of the 595 bp alignment) and 91 unique haplotypes were observed in the 359 individuals scored from the *nad4* gene of the 18 *B. minax* populations. This indicated that *B. minax* had a high level of genetic diversity. These populations also showed a discrete distribution in both the scatter plots of genetic versus geographical distance for pairwise population comparisons and the median-joining network of haplotypes, which revealed the strong genetic structure of *B. minax*.

## 1. Introduction

The Chinese citrus fly, *Bactrocera minax* (Enderlein) (Diptera: Tephritidae), is an important pest of citrus fruits in Asia [1,2,3]. Infestations cause serious economic losses and also trigger international trade embargoes. *B. minax* was first reported in Sikkim, India in 1920, and the first infestation in China was reported in Chongqing in 1940. This species was formally named the Chinese citrus fly in 1955 to distinguish it from the Japanese orange fly, *B.* (*Tetradacus*) *tsuneonis* (Miyake) [4]. There have been records of *B. minax* in 134 counties and cities of nine provinces in China so far, and the geographic distribution of the pest continues to expand.

*B. minax* is considered unique among the members of family Tephritidae in being univoltine, i.e., one generation per year [5,6]. The larvae of *B. minax* live and feed inside host fruit, making effective pest control difficult. Most published research on *B. minax* has focused on the basic biology, ecology, and behavior [6], and phylogenetic studies [7,8]. Population genetics study would provide theoretical basis for analyzing evolutionary potential and environmental adaptability as well as to make integrated prevention and control measures. In 2011 and 2012, four mitochondrial DNA (mtDNA) genes (*cox1*; *nad1*, *cytb*, and *nad5*) were used to infer two main invasion routes and origin of the oriental fruit fly, *B. dorsalis* (Hendel) [9,10]. Thereafter, more targeted methods were developed based on these findings for a preventive of the oriental fruit fly. Relatively, data about expansion processes of *B. minax* is scarce despite the current and potential risk from this species.

Animal mtDNA is a favorite molecule for evolutionary studies due to several special characteristics, such as simple sequence organization, maternal inheritance, and absence of recombination [11]. Among the mitochondrial genes, *nad4* is regarded as a very useful marker owing to its high rate of evolution and rich polymorphism [12]. In this paper, the genetic structure of *B. minax* in Mainland China was investigated through analysis of molecular variations in a fragment of the mtDNA gene *nad4*, and we also studied genetic diversity among different geographic populations of *B. minax* in China.

## 2. Materials and Methods

### 2.1. Sampling

**Figure 1:**
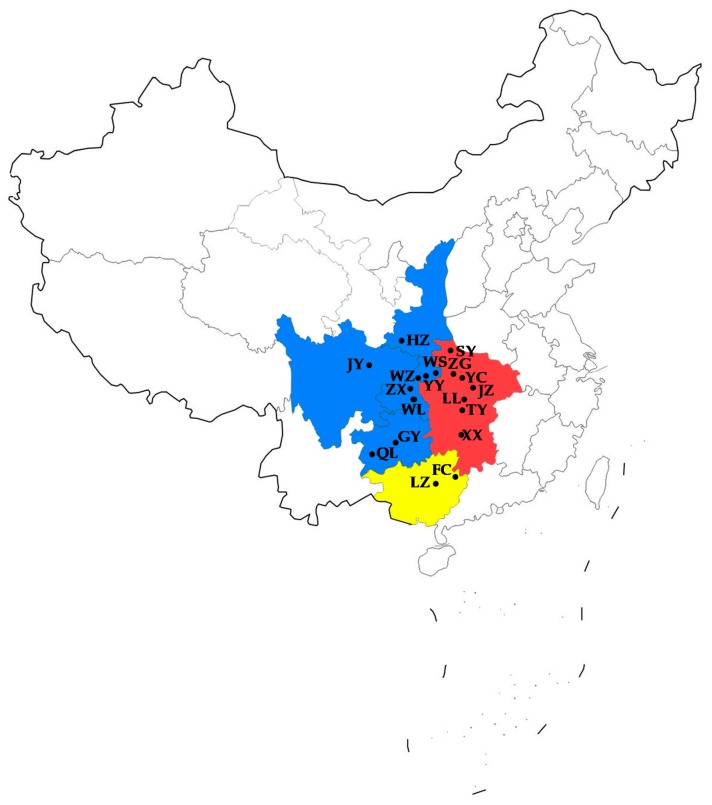
Sample collection sites of *Bactrocera minax* in China. Blue, Western China; Red, Central China; Yellow, Southern China.

A total of 359 *B. minax* adults were trapped in citrus orchards through pheromone traps at 18 locations in China (Table 1 and Figure 1). No specific permissions were required to collect from these locations, which were not privately owned or protected in any way, and our study did not involve endangered or protected species. The samples were preserved in 95% ethanol at 4 °C prior to DNA extraction.

### 2.2. DNA Extraction and Amplification

Total DNA was extracted using DNeasy Blood and Tissue Kits (QIAGEN, Venlo, Netherlands) from the thorax of each individual. A fragment of the mtDNA gene *nad4* (639 bp595) was amplified as described by Zhang [13], using primer pairs *nad4*-F (5′-ACAAAACAAACCTGACGAAC-3′) and *nad4*-R (5′-TAGTAGAATGAATCTTTTTATA-3′). Each PCR was performed in a volume of 25 µL containing 80 ng of total genomic DNA, 0.25 µM of each primer, 1.5 mM MgCl_2_, 10 × PCR buffer, 0.2 mM dNTPs, and 1.25 U Taq DNA polymerase (Promega, Fitchburg, WI, USA). The temperature profile included an initial denaturation at 95 °C for 5 min followed by 35 cycles of 94 °C for 45 s, 48 °C for 60 s, and 72 °C for 45 s, and a final extension step of 72 °C for 10 min. PCR products were subsequently purified and sequenced in both forward and reverse directions (Invitrogen, Shanghai, China).

### 2.3. Data Analysis

Sequence alignment and identification of unique haplotypes were performed using ClustalX ver. 2.1 [14], and then deposited into GenBank for public release. Demographic history analyses, including Tajima’s *D* test [15], Fu’s *F* statistic [16], and mismatch distribution, were carried out using ARLEQUIN ver. 3.11 [17,18]. Descriptive statistics of genetic diversity were calculated with DNAsp ver. 5.0 [19], including polymorphic sites, number and constitution of haplotypes, number of variable sites, haplotype diversity, nucleotide diversity, and average number of nucleotide differences.

The 18 geographic populations were grouped using SAMOVA ver. 1.0 based on sequence information and coordinate data [20]. The most likely number of groups (*K*) was the value that maximized *F_CT_*. ARLEQUIN ver. 3.11 was used to perform hierarchical analysis of molecular variance (AMOVA) based on the grouping results from SAMOVA, including comparisons among groups, among populations, and within populations.

Pairwise genetic distances between populations were estimated using MEGA ver. 5.05 [21], and then the phylogenetic trees inferred via neighbor-joining (NJ) and maximum-likelihood (ML) methods were reconstructed with 1000 bootstrap replicates using MEGA ver. 5.05 and PhyML ver. 3.0 [22], respectively. Median-joining networks for inferring the evolutionary relationships among haplotypes of the 18 geographic populations were constructed based on statistical parsimony using Network ver. 4.6 [23,24].

## 3. Results

### 3.1. Genetic Diversity

Of 359 individuals scored from the *nad4* gene, 93 variable sites (15.6% of the 595 bp alignment) and 91 unique haplotypes were observed (GenBank accession numbers KF310532-KF310890). The *nad4* protein-coding region began at the first site of the sequence. Like the two mitochondrial genes *16S rDNA* and *COI* (A+T biased) [25], the base composition of the *nad4* was also biased toward A+T (65.5%). The populations showed a high level of haplotype diversity (*h*) which ranged from 0.386 at WZ to 0.928 at WL, nucleotide diversity (*π*) ranged from 0.001 at JZ to 0.009 at JY, and the average number of nucleotide differences (*k*) ranged from 0.837 at JZ to 5.584 at JY, except for the ZX population (*h* < 0.001, *π* < 0.001, *k* < 0.001) (Table 2). Only 15 of the 91 haplotypes were shared by at least two populations; the most frequent haplotype, H32, was shared by 10 populations, followed by H8 which was shared by eight populations.

### 3.2. Population Genetic Structure

The *F_CT_* values suggested an optimum of three groups for the 18 populations, as follows: (i) Central China (CC, including the SY, ZG, YC, JZ, XX, TY and LL population); (ii) Western China (WC, including the QL, GY, WL, ZX, WZ, YY, WS, HZ and JY population); and (iii) Southern China (SC, including the FC and LZ population). Results of AMOVA showed that the largest percentage of variation (46.15%) occurred among groups (Table 3), while 15.84% of variation occurred among populations within groups and 38.01% within populations. Meanwhile, the fixation index among groups was almost double that among populations within groups (*F_CT_* = 0.462, *p* < 0.01; *Fsc* = 0.294, *p* < 0.01), indicating that there may be factors limiting the gene flow among regions.

Pairwise *F_ST_* values among populations ranged from −0.018 (between the FC and LZ population) to 0.796 (between the WZ and ZX population), while the average values ranged from 0.277 (from the other populations to LL) to 0.612 (from the other populations to YC) (Table S1). Pairwise *F_ST_* values among groups ranked as follows: 0.053 (between SC and WC < 0.211 (between CC and WC) < 0.315 (between CC and SC) (Table 4).

### 3.3. Estimation of Migration Rates

The migration rates were estimated using Bayesian inference in Migrate ver. 3.5.1, which revealed high effective immigration rates per generation between regions (Table S2). Unidirectional estimates of *M* ranged from 47.0 (WZ→ZG) to 838.3 (LL→JZ), and no asymmetrical gene flow was found. The gene flow values among populations ranged from 0.156 (WL→ZX) to 21.508 (TY→ZG), the immigration rates varied from 0.322 of ZX to 10.006 of TY, and the emigration rates from populations ranged from 1.561 of FC to 3.319 of SY. The immigration rates of ZG, TY, and LL populations (8.859, 10.006, and 9.256, respectively) were significantly higher than the emigration rates of these three populations, and were also significantly higher than immigration rates of other populations.

The results of gene flow among the three clusters based on *nad4* sequences showed that the SC cluster had the lowest immigration rate (1.175) and the highest emigration rate (22.785), and the situation (the immigration rate < the emigration rate) was opposite to the CC and WC clusters (Table 5).

### 3.4. Phylogenetic Analysis

#### 3.4.1. Phylogenetic Trees Construction

**Figure 2:**
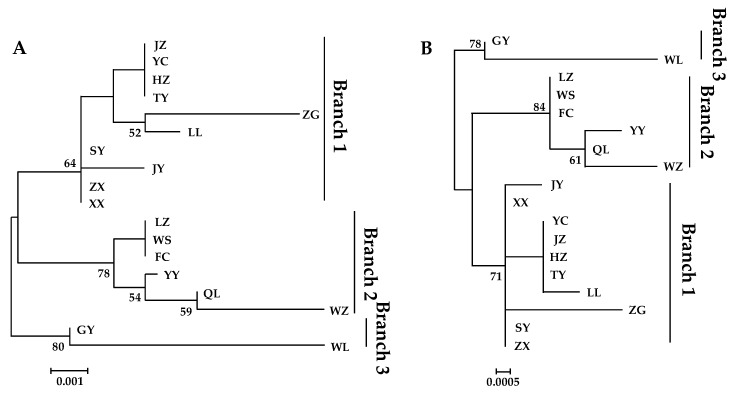
Phylogenetic analyses of *nad4* sequence from 18 *Bactrocera minax* populations. (**A**) Phylogenetic tree of *nad4* sequence using the neighbor-joining method estimated with MEGA v. 5.05. (**B**) Phylogenetic tree of *nad4* sequence using the maximum-likelihood method estimated with PhyML v. 3.0. Bootstrap value was indicated at each node.

Both phylogenetic trees constructed by NJ and ML methods were divided into three branches with the same classification based on the *nad4* sequence of 18 populations (Figure 2A,B). However, these branches were not consistent with the SAMOVA groups. The first branch included all the CC populations as well as three WC populations (HZ, JY, and ZX). All the SC populations and four WC populations (QL, WZ, YY, and WS) comprised the second branch. The remaining two WC populations (GY, and WL) fell into the third branch. One possible reason for the different grouping results is that *B. minax* populations have resulted from multiple invasions.

#### 3.4.2. Median-Joining Network of Haplotypes

**Figure 3:**
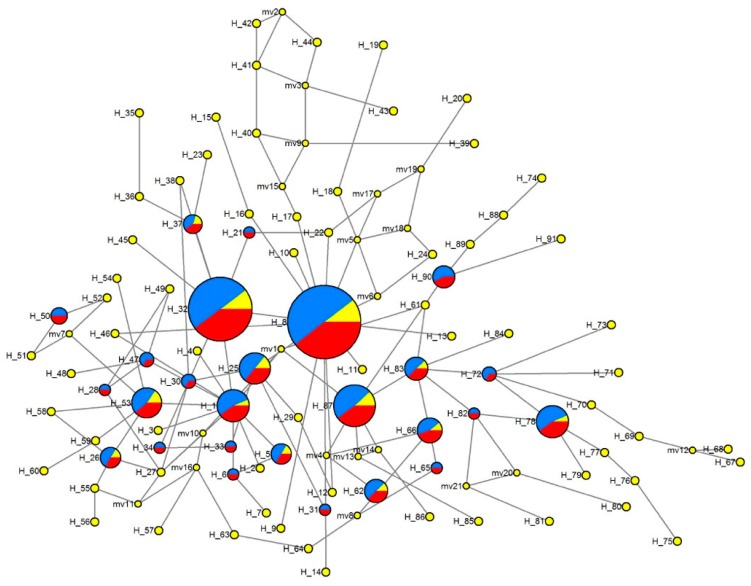
Median-joining network of haplotypes. Blue, Western China; red, Central China; yellow, Southern China.

The MJ network showed that some haplotypes at higher frequency (e.g., H8, H32, H87, H78, H25, and H1) were located centrally, with rare haplotypes connected to them through few mutations. Six haplotypes (H1, H8, H25, H32, H66, and H87) were shared by at least two groups but only two were shared by all three groups (H32, and H87) (Figure 3).

### 3.5. Demographic Analysis

Based on the *nad4* gene, neutrality test results showed that Tajima’s *D* values were significantly negative for all *B. minax* populations pooled together and the CC+WC clusters (ranging from −1.979 to −1.764, *p* < 0.01), but not for the SC cluster (0.534, *p* > 0.05). Tajima’s *D* values ranged from −2.398 (*p* < 0.01) of WZ to 1.639 of HZ (*p* > 0.05). The values of Tajima’s *D* were significantly different among these populations (Table 2). Fu’s *F_s_* values were significantly negative for all populations pooled together and for each group individually (ranging from −26.622 to −24.928, *p* < 0.01) and also significantly negative in each population (ranging from −34.028 of JZ to −15.700 of WL), except for the ZX population (34.028, *p* > 0.05) (Table 3).

**Figure 4:**
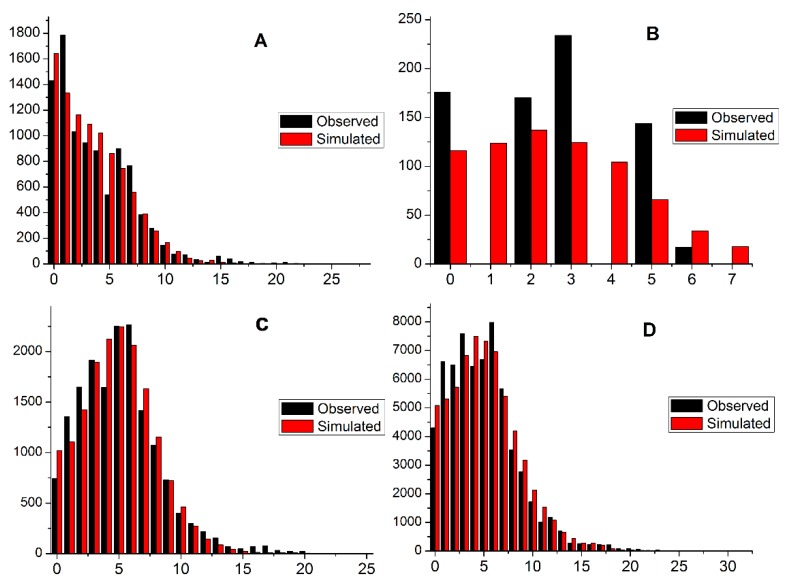
Observed and simulated mismatch distributions of all *Bactrocera minax* populations. (**A**) Central China; (**B**) Southern China; (**C**) Western China; (**D**) All populations.

The unimodal mismatch distributions based on *nad4* data from the 18 populations and each population group revealed a sudden demographic expansion (Figure 4). The expansion (coalescence) time under the sudden expansion assumption in mutation-generations (*τ*) ranged from 0.000 (ZX) to 10.309 (YC) (Table 2). To estimate coalescence time in the absence of a molecular clock calibration for *B. minax*, we used a mutation rate (*μ*) of 1.7 × 10^−8^ per year for mitochondrial genes [26]. The oldest coalescence time for *nad4* gene of *B. minax* was in the YC population with about 448,000 years, XX and JZ populations were approximately 10,000 (*τ* = 0.066) and 170 (*τ* = 0.004) years old. And the most special was the ZX population with no expansion history (*τ* = 0.000), which may be newly established.

## 4. Discussion

A species’ genetic resources exist at two fundamental levels, genetic differences among individuals within local populations and those among populations [27]. All the *B. minax* populations had moderately high levels of genetic diversity except the ZX population with only one haplotype, which may represent a newly-colonized site. Based on the pairwise *F_ST_* values for *nad4* sequences, the 18 *B. minax* populations were highly genetically differentiated. Meanwhile, the AMOVA results of the 18 populations showing 46.15% variation among groups, indicating a strong population genetic structure of *B. minax* in Mainland China. Moreover, the scatter plots of genetic versus geographical distances for pairwise population comparisons and the median-joining network of haplotypes also exhibit a discrete distribution and support the same conclusion. Combining the differences among SAMOVA with phylogeny analysis and pairwise *F_ST_* values among populations, we hypothesize that *B. minax* may have invaded these areas on multiple separate occasions. The results of AMOVA and haplotypes phylogeny analyses, based on *nad4* sequence data, showed limited gene flow among regions and suggested that the Yangtze River, Nanling Mountains, and Wushan Mountains may have acted as substantial barriers to gene flow.

Four main factors promote biological evolution: mutation, gene flow, random genetic drift, and natural selection [28]. Levels of gene flow depend not only on the distances among populations, but also on the nature of the surrounding landscapes [29,30,31,32]. Under equilibrium conditions, gene flow offsets the effects of genetic drift, and pairwise *F_ST_* estimates will increase with geographical distance [33]. The values of gene flow from other populations to ZX were much lower than others (130.3 < M < 457.0). There should be much citrus trade to the cities of ZG, TY, and LL from the other 15 sites. The immigration and emigration rates were very unequal in the SC cluster (emigration rate >> immigration rate), suggesting that the SC cluster may be a major dispersal source for *B. minax* in mainland China. In contrast, the immigration rate (21.591) was much higher than the emigration rate (3.199) in the WC cluster, while the immigration rate (8.696) was basically symmetrical with the emigration rate (5.447) in the CC cluster (Table 5). The gene flow analysis supports the view that the Yangtze River, Nanling Mountains, and Wushan Mountains have acted as substantial barriers to gene flow. There were no strong isolation-by-distance relationships among *B. minax* populations [34], the reason for which may be related to the frequent citrus trade between the cities and insect movement aided by human commerce. A positive Tajima’s *D* means low levels of low-frequency polymorphisms, implying a decrease in population size or selection of balancing; meanwhile, a negative Tajima’s *D* means excess levels of low-frequency polymorphisms, implying an expansion in population size or selection of purifying. The same principle holds for Fu’s *F_s_* test [35,36]. The rate of random walk will depend on population size (Wright’s “effective breeding size”) and the mating structure. The smaller the population, the greater the random variation, and thus the faster the random walk [37]. Based on the neutrality test of *B. minax nad4* sequences, the significantly negative Tajima’s *D* and Fu’s *F_s_* values suggest an absence of historical population bottlenecks and subsequent population expansion and an excess of low frequency polymorphisms. Besides, the negative fixation index (*F_ST_* = −0.018, −0.004) indicated that there was still excess heterozygosity in some *B. minax* populations. Moreover, the unimodal mismatched distributions of the *nad4* gene within the 18 *B. minax* populations meant a population expansion in agreement with the negative Tajima’s *D*, and reflected the main coalescence depth of the haplotypes which is ultimately traces to a single ancestor.

In 1940, *B. minax* was first recorded in mainland China. Since then, *B. minax* has continued to spread. It was found in five provinces in 1957, six in 1966, eight in 1995, and nine in 2006. The centers of origin and the diffusion paths of *B. minax* are unknown. In this study, it was found that the oldest coalescence time for *nad4* gene in *B. minax* was about 448,000 years ago, and the ZX population may be newly established with no expansion history. As to the demographic history the major dispersal routes of *B. minax* into Mainland China, we would need more loci and more samples.

## 5. Conclusions

We collected 359 *B. minax* individuals from 18 localities in China, and used a mitochondrial DNA gene fragment *nad4* to investigate the genetic diversity and population genetic structure of this species. Eighteen *B. minax* populations were divided by phylogenetic analyses and statistical parsimony haplotype networks into three branches: a Central China (CC) branch, a Western China (WC) branch, and a Southern China (SC) branch. A total of 93 variable sites and 91 unique haplotypes were observed in the 359 nucleotides scored from the *nad4* gene, which indicated that *B. minax* had a high level of genetic diversity. These populations also showed a discrete distribution in both the scatter plots of genetic versus geographical distance for pairwise population comparisons and the median-joining network of haplotypes, which revealed the strong genetic structure of *B. minax*.

## Figures and Tables

**Table 1:** Sampling information of *Bactrocera minax* specimens.

Location (City, Province)	Abbreviation	Number of Individuals	Longitude	Latitude
Shiyan, Hubei	SY	21	110°28′(E)	32°23′(N)
Zigui, Hubei	ZG	21	110°58′(E)	30°49′(N)
Yichang, Hubei	YC	19	111°19′(E)	30°46′(N)
Jingzhou, Hubei	JZ	20	112°14′(E)	30°20′(N)
Xiangxi, Hunan	XX	16	109°44′(E)	28°18′(N)
Taoyuan, Hunan	TY	21	111°28′(E)	28°54′(N)
Linli, Hunan	LL	20	111°38′(E)	29°26′(N)
Fuchuan, Guangxi	FC	19	111°16′(E)	24°38′(N)
Luzhai, Guangxi	LZ	20	109°43′(E)	24°28′(N)
Qinglong, Guizhou	QL	20	105°12′(E)	25°50′(N)
Guiyang, Guizhou	GY	21	106°39′(E)	26°24′(N)
Wulong, Chongqing	WL	18	107°45′(E)	29°19′(N)
Zhongxian, Chongqing	ZX	21	108°01′(E)	30°18′(N)
Wanzhou, Chongqing	WZ	19	108°24′(E)	30°48′(N)
Yunyang, Chongqing	YY	20	108°41′(E)	30°55′(N)
Wushan, Chongqing	WS	23	109°52′(E)	31°04′(N)
Hanzhong, Shanxi	HZ	20	107°01′(E)	33°04′(N)
Jiangyou, Sichuan	JY	20	104°44′(E)	31°46′(N)

**Table 2:** Parameters of genetic diversity of *nad4*.

Population	*S*	*h*	*k*	*π*	Tajima’s *D*	Fu’s *F_S_*	*τ*	*P*_SSD_
SY	4	0.724	1.095	0.002	0.787	−27.046 **	3.742	0.180
ZG	14	0.924	3.824	0.007	0.157	−24.443 **	4.926	0.390
YC	7	0.526	1.076	0.002	−1.558 *	−20.489 **	10.309	0.480
JZ	5	0.432	0.837	0.001	−1.243	−34.028 **	0.004	0.000
XX	5	0.425	1.225	0.002	−0.274	−28.882 **	0.066	0.000
TY	15	0.810	2.457	0.004	−1.535 *	−26.755 **	0.525	0.000
LL	23	0.800	3.105	0.005	−1.778 *	−23.195 **	0.352	0.000
FC	6	0.708	2.117	0.003	0.755	−26.845 **	3.414	0.040
LZ	9	0.795	2.937	0.005	0.542	−25.571 **	4.621	0.040
QL	14	0.837	3.474	0.006	−0.849	−21.614 **	6.227	0.120
GY	11	0.886	2.405	0.004	−0.355	−25.177 **	3.818	0.170
WL	21	0.928	5.327	0.009	−0.499	−15.610 **	5.787	0.410
ZX	0	0.000	0.000	0.000	0.000	34.028	0.000	0.000
WZ	28	0.386	3.146	0.005	−2.398 **	−23.178 **	3.000	0.160
YY	16	0.868	2.832	0.005	−1.383	−25.864 **	2.236	0.670
WS	11	0.818	2.996	0.005	0.018	−26.357 **	4.125	0.340
HZ	2	0.479	0.958	0.002	1.639	−34.028 **	2.761	0.160
JY	22	0.837	5.584	0.010	−0.215	−17.651 **	6.268	0.090
All	93	0.917	4.217	0.007	−1.979 **	−24.928 **	4.287	0.860
CC					−1.923 **	−25.881 **	7.143	0.910
SC					0.534	−26.622 **	3.639	0.080
WC					−1.764 **	−25.194 **	4.504	0.760

*S*, number of variable sites; *h*, haplotype diversity; *k*, average number of nucleotide differences; *π*, nucleotide diversity; neutrality tests: Tajima’s *D*, Fu’s *F* statistic, and expansion (coalescence) time under the sudden expansion assumption in mutation-generations (*τ*); *P*_SSD_: *p* value for sum of squared deviations (SSD). * *p* < 0.05, ** *p* < 0.01.

**Table 3:** Partitioning of genetic variation at different hierarchical levels.

Locus Analyzed	Source of Variation	*df*.	Sum of Squares	Variance Components	Percentage of Variation	Fixation Indices
*nad4*	Among groups	2	202.397	1.740 Va	46.15	*F_CT_* = 0.462 **
	Among populations within groups	15	216.748	0.597 Vb	15.84	*F_SC_* = 0.294 **
	Within populations	341	2588.323	1.433 Vc	38.01	*F_ST_* = 0.620 **

** *p* < 0.01.

**Table 4:** The genetic differentiation index *F_ST_* values of *nad4* population pairwise comparisons.

Group	CC	SC	WC
CC	—		
SC	0.315 **	—	
WC	0.211 **	0.053 **	—

CC, Central China; SC, Southern China; WC, Western China. ** *p* < 0.01.

**Table 5:** Estimates of gene flow among three groups of *Bactrocera minax* populations.

	Group	*θ*	*M*		
			CC→*i*	SC→*i*	WC→*i*
*nad4*	CC	0.032 (0.013–0.061)	—	382.3 (64.0–794.7)	158.3 (0.0–589.3)
	SC	0.004 (0.000–0.010)	298.3 (0.0–825.3)	—	373.0 (60.0–798.0)
	WC	0.072 (0.034–0.100)	137.7 (0.0–260.0)	462.3 (119.3–878.0)	—

CC, Central China; SC, Southern China; WC, Western China. *θ* effective population size before expansion, M, mutation-scaled effective immigration rate. 95% highest probability density intervals are shown in parentheses.

## Supplementary Materials

The following are available online at https://www.mdpi.com/2075-4450/10/8/236/s1, Table S1: Pairwise comparisons between *F_ST_* values of *nad4* gene fragments from 18 *Bactrocera minax* populations. Table S2: Estimates of gene flow between 18 *Bactrocera minax* populations.
