# Supplementary material for: Population Genetics of Bactrocera minax (Diptera: Tephritidae) in China Based on nad4 Gene Sequence
Source: Insects. 2019 Aug 2;10(8):236. doi: 10.3390/insects10080236 (PMC6723541; doi:10.3390/insects10080236)
Supplement: Supplementary file 1 [file insects-10-00236-s001.zip › insects-538025-SI.pdf]

Table S1. Pairwise comparisons between F_{ST} values of *nad4* gene fragment from 18 *Bactrocera minax* populations.

Population	SY	ZG	YC	JZ	XX	TY	LL	FC	LZ	QL	GY	WL	ZX	WZ	YY	WS	HZ	JY
SY	—																	
ZG	0.192**	—																
YC	0.592**	0.463**	—															
JZ	0.204**	0.169**	0.629**	—														
XX	0.384**	0.206**	0.637**	0.441**	—													
TY	0.056*	0.109**	0.549**	0.033	0.238**	—												
LL	0.004	0.107**	0.517**	0.050*	0.203**	-0.004	—											
FC	0.502**	0.381**	0.657**	0.541**	0.254*	0.425**	0.362**	—										
LZ	0.458**	0.357**	0.630**	0.481**	0.226**	0.388**	0.334**	-0.018	—									
QL	0.293**	0.267**	0.545**	0.335**	0.232**	0.253**	0.228**	0.247**	0.238**	—								
GY	0.386**	0.367**	0.619**	0.478**	0.331**	0.374**	0.331**	0.317**	0.300**	0.035*	—							
WL	0.388**	0.330**	0.566**	0.414**	0.247**	0.350**	0.309**	0.142**	0.141**	0.117**	0.135**	—						
ZX	0.502**	0.253**	0.712**	0.643**	0.150*	0.315**	0.277**	0.547**	0.463**	0.368**	0.474**	0.409**	—					
WZ	0.720**	0.649**	0.744**	0.761**	0.681**	0.693**	0.639**	0.550**	0.530**	0.570**	0.617**	0.480**	0.796**	—				
YY	0.659**	0.586**	0.717**	0.716**	0.602**	0.634**	0.567**	0.399**	0.391**	0.470**	0.524**	0.358**	0.752**	0.198**	—			
WS	0.495**	0.411**	0.647**	0.517**	0.323**	0.438**	0.378**	0.064*	0.060	0.272**	0.343**	0.161**	0.534**	0.455**	0.252**	—		
HZ	0.228**	0.165**	0.630**	0.045	0.313**	0.062*	0.075*	0.443**	0.393**	0.249**	0.387**	0.332**	0.528**	0.735**	0.679**	0.443**	—	
JY	0.418**	0.335**	0.542**	0.420**	0.249**	0.367**	0.330**	0.165**	0.177**	0.272**	0.326**	0.214**	0.398**	0.468**	0.363**	0.208**	0.367**	—

Population abbreviations are defined in Table 1. * $P < 0.05$, ** $P < 0.01$

Table S2. Estimates of gene flow between 18 *Bactrocera minax* populations.

Population	θ	M																	
		SY→*i*	ZG→*i*	YC→*i*	JZ→*i*	XX→*i*	TY→*i*	LL→*i*	FC→*i*	LZ→*i*	QL→*i*	GY→*i*	WL→*i*	ZX→*i*	WZ→*i*	YY→*i*	WS→*i*	HZ→*i*	JY→*i*
SY	0.003	—	451.7 (110.0–563.3)	401.0 (36.0–750.7)	583.7 (234.0–1000.0)	210.3 (0.0–610.7)	322.3 (0.0–844.7)	510.3 (109.3–1000.0)	430.3 (80.0–836.7)	281.7 (0.0–754.0)	499.7 (120.7–944.0)	427.7 (38.0–808.7)	250.3 (0.0–597.3)	282.3 (0.0–518.0)	391.7 (0.0–825.3)	333.7 (0.0–756.7)	381.7 (15.3–784.7)	621.0 (301.3–988.0)	499.0 (0.0–470.1)
ZG	0.040	319.7 (30.0–487.3)	—	131.0 (0.0–379.3)	219.0 (0.0–429.3)	268.3 (0.0–561.3)	533.7 (290.0–982.7)	99.0 (0.0–292.0)	180.3 (20.7–580.7)	180.3 (0.0–488.7)	300.3 (0.0–704.7)	317.0 (2.7–743.3)	217.0 (0.0–722.7)	185.0 (0.0–538.7)	47.0 (0.0–182.0)	117.7 (0.0–378.0)	172.3 (0.0–548.7)	295.7 (0.0–663.3)	153.7 (0.0–518.0)
YC	0.002	593.7 (224.7–990.7)	143.0 (0.0–442.7)	—	188.3 (0.0–691.3)	141.7 (0.0–587.3)	257.7 (0.0–668.0)	299.0 (0.0–690.0)	351.0 (0.0–542.0)	335.7 (0.0–769.3)	411.7 (25.3–643.3)	307.7 (2.7–571.3)	167.0 (0.0–420.0)	317.7 (0.0–643.3)	301.7 (3.3–718.0)	389.0 (0.0–530.7)	141.0 (0.0–451.3)	469.7 (114.7–864.7)	415.7 (0.0–429.4)
JZ	0.001	501.3 (0.0–543.0)	610.3 (316.0–1000.0)	476.3 (130.0–781.3)	—	715.7 (340.0–995.3)	357.7 (0.0–761.3)	838.3 (562.0–1000.0)	622.3 (496.7–1000.0)	605.7 (398.0–1000.0)	621.7 (224.7–1000.0)	451.0 (0.0–462.7)	733.7 (320.7–1000.0)	627.0 (444.0–993.3)	293.0 (0.0–712.7)	477.1 (0.0–529.0)	470.3 (0.0–838.7)	641.0 (220.7–1000.0)	481.2 (0.0–492.3)
XX	0.001	475.0 (0.0–531.3)	631.0 (351.3–996.7)	559.7 (202.0–1000.0)	715.0 (528.7–1000.0)	—	578.3 (185.3–990.7)	519.0 (92.7–852.0)	665.7 (472.0–995.3)	667.7 (296.0–1000.0)	566.3 (129.3–1000.0)	325.0 (0.0–600.0)	665.7 (304.0–1000.0)	719.7 (390.0–1000.0)	537.6 (516.3–993.3)	496.3 (122.0–916.0)	534.3 (526.6–978.7)	476.2 (445.0–1000.0)	387.7 (0.0–752.0)
TY	0.027	804.3 (488.7–1000.0)	435.0 (134.7–757.3)	557.0 (154.0–989.3)	554.3 (184.7–999.3)	650.3 (288.0–984.0)	—	441.7 (68.7–697.3)	108.3 (0.0–462.7)	171.7 (0.0–451.3)	141.7 (0.0–457.3)	345.0 (0.0–589.3)	211.7 (0.0–549.3)	246.3 (0.0–553.3)	280.3 (0.0–6–7.3)	510.2 (0.0–521.0)	494.3 (32.7–888.7)	203.0 (0.0–443.3)	182.3 (0.0–495.3)
LL	0.021	395.7 (0.0–784.7)	331.7 (0.0–706.7)	225.7 (0.0–634.7)	341.7 (0.0–721.3)	111.0 (0.0–349.2)	508.3 (367.3–1000.0)	—	172.3 (0.0–762.0)	492.3 (88.7–718.7)	367.7 (4.0–790.0)	603.7 (502.0–998.0)	547.0 (103.0–992.0)	452.3 (0.0–888.0)	542.3 (130.0–900.7)	473.7 (246.7–745.3)	322.3 (0.0–763.3)	697.7 (396.7–990.7)	767.7 (399.3–1000.0)
FC	0.001	653.7 (337.3–1000.0)	625.0 (261.3–994.0)	621.0 (120.7–978.0)	543.5 (536.3–1000.0)	679.7 (292.0–996.0)	603.0 (241.0–1000.0)	510.3 (90.7–679.3)	—	603.7 (230.0–984.0)	707.7 (494.7–1000.0)	489.6 (459.0–989.3)	579.0 (535.3–1000.0)	701.0 (300.7–1000.0)	739.7 (285.3–1000.0)	377.0 (0.0–590.0)	472.3 (0.0–844.7)	450.5 (414.3–1000.0)	652.3 (280.0–1000.0)
LZ	0.001	582.3 (392.7–978.0)	398.3 (0.0–418.9)	459.0 (0.0–491.0)	671.0 (380.0–1000.0)	558.3 (502.0–983.3)	723.0 (430.0–1000.0)	655.0 (384.7–1000.0)	581.7 (203.3–994.0)	—	473.0 (140.0–810.7)	375.0 (0.0–780.7)	659.0 (323.0–1000.0)	389.7 (0.0–552.7)	542.3 (480.0–1000.0)	659.7 (510.0–1000.0)	487.2 (0.0–495.0)	540.3 (514.1–1000.0)	394.3 (0.0–399.3)
QL	0.006	676.3 (490.7–998.7)	627.7 (532.7–994.0)	583.7 (152.7–1000.0)	337.7 (0.0–787.3)	639.0 (448.7–1000.0)	687.0 (325.3–1000.0)	622.3 (292.0–976.0)	199.0 (0.0–612.0)	491.7 (108.7–907.3)	—	481.7 (0.0–493.0)	115.0 (0.0–484.0)	136.3 (0.0–426.7)	113.0 (0.0–386.0)	248.3 (0.0–691.3)	271.0 (0.0–744.0)	140.3 (0.0–655.3)	253.7 (0.0–690.7)

Population	θ	M																	
		SY→*i*	ZG→*i*	YC→*i*	JZ→*i*	XX→*i*	TY→*i*	LL→*i*	FC→*i*	LZ→*i*	QL→*i*	GY→*i*	WL→*i*	ZX→*i*	WZ→*i*	YY→*i*	WS→*i*	HZ→*i*	JY→*i*
GY	0.0026	277.0 (0.0–694.7)	364.3 (0.0–708.0)	240.3 (0.0–484.0)	683.0 (391.3–1000.0)	272.3 (0.0–762.0)	283.7 (0.0–361.3)	481.0 (0.0–844.0)	354.3 (0.0–803.3)	235.7 (0.0–630.0)	237.7 (0.0–598.7)	—	469.7 (40.7–827.3)	685.0 (300.0–988.7)	337.0 (22.7–603.3)	453.0 (0.0–845.3)	490.3 (122.0–811.3)	265.0 (0.0–522.7)	600.3 (497.3–989.3)
WL	0.003	555.7 (527.8–1000.0)	631.7 (229.3–1000.0)	259.0 (0.0–666.0)	578.3 (188.7–1000.0)	256.3 (0.0–774.0)	568.5 (0.0–602.3)	498.8 (0.0–581.0)	479.0 (95.3–591.3)	632.3 (492.0–1000.0)	593.7 (287.3–1000.0)	759.7 (462.7–1000.0)	—	189.7 (0.0–545.3)	323.7 (0.0–528.7)	428.3 (0.0–429.9)	215.7 (0.0–704.7)	170.3 (0.0–464.7)	218.3 (0.0–629.3)
ZX	0.001	229.7 (0.0–556.0)	346.3 (0.0–550.0)	331.0 (16.7–655.3)	188.3 (0.0–505.3)	175.7 (0.0–504.7)	457.0 (110.7–817.3)	255.7 (0.0–689.3)	281.7 (0.0–700.0)	231.7 (0.0–671.3)	139.7 (0.0–430.7)	349.0 (0.0–783.3)	130.3 (0.0–436.7)	—	378.3 (4.7–751.3)	337.7 (0.0–759.3)	377.0 (11.3–824.7)	200.3 (0.0–627.3)	157.0 (0.0–460.0)
WZ	0.002	143.7 (0.0–396.7)	302.3 (0.0–599.3)	223.7 (0.0–586.0)	228.3 (0.0–794.7)	258.3 (0.0–674.0)	182.3 (0.0–657.3)	131.0 (0.0–414.7)	307.7 (0.0–660.7)	133.7 (0.0–375.3)	278.3 (0.0–668.0)	244.3 (0.0–601.3)	324.3 (0.0–691.3)	275.0 (0.0–543.3)	—	429.7 (0.0–603.3)	598.3 (328.7–990.0)	377.0 (0.0–432.0)	412.3 (18.0–500.0)
YY	0.002	254.3 (0.0–627.3)	722.3 (322.0–1000.0)	214.3 (0.0–548.0)	299.7 (0.0–681.3)	589.0 (213.3–1000.0)	566.3 (386.7–1000.0)	722.3 (321.3–1000.0)	588.3 (183.3–975.3)	513.0 (504.5–1000.0)	604.8 (0.0–684.3)	531.7 (517.0–988.7)	481.0 (132.7–714.0)	726.3 (402.7–998.7)	662.3 (276.0–1000.0)	—	297.7 (0.0–595.3)	517.0 (0.0–572.3)	353.0 (0.0–723.3)
WS	0.002	720.3 (408.0–1000.0)	757.7 (531.3–1000.0)	445.0 (0.0–463.7)	606.3 (426.0–1000.0)	662.3 (218.7–1000.0)	333.7 (0.0–418.7)	603.0 (203.3–972.7)	418.3 (0.0–479.3)	718.3 (389.3–1000.0)	623.7 (334.7–1000.0)	664.3 (295.3–1000.0)	358.3 (0.0–689.3)	662.3 (206.7–985.3)	727.7 (280.7–1000.0)	482.4 (0.0–498.3)	—	357.0 (0.0–780.0)	495.7 (115.3–967.3)
HZ	0.001	570.3 (555.5–996.7)	528.3 (139.3–965.3)	463.6 (0.0–488.3)	517.7 (154.7–1000.0)	762.3 (465.3–1000.0)	547.4 (0.0–564.3)	507.9 (0.0–554.3)	635.7 (350.7–991.3)	274.3 (0.0–614.7)	459.0 (0.0–472.3)	353.0 (0.0–490.0)	745.0 (460.7–1000.0)	566.3 (203.3–1000.0)	478.3 (126.7–622.7)	471.7 (14.7–662.7)	595.0 (203.0–1000.0)	—	369.7 (0.0–708.7)
JY	0.002	575.7 (323.3–972.0)	572.3 (155.3–974.7)	219.0 (0.0–680.0)	445.0 (41.3–898.7)	566.0 (0.0–611.7)	772.3 (362.0–1000.0)	277.7 (13.3–559.3)	741.0 (517.3–1000.0)	411.0 (0.0–1000.0)	517.0 (475.9–1000.0)	590.3 (316.0–1000.0)	262.3 (0.0–721.3)	652.3 (170.0–995.3)	501.7 (74.0–890.0)	441.2 (0.0–454.3)	639.7 (236.7–10000.0)	569.7 (300.7–996.7)	—

Population abbreviations are defined in Table 1. θ, mutation-scaled effective population size. M, mutation-scaled effective immigration rate. 95% highest probability density intervals are shown in parentheses.
